# Vitamin A status in healthy women eating traditionally prepared spirulina (*Dihé*) in the Chad Lake area

**DOI:** 10.1371/journal.pone.0191887

**Published:** 2018-01-29

**Authors:** Imar Djibrine Soudy, Régine Minet-Quinard, Alhadj Djidda Mahamat, Hadjé Fatimé Ngoua, Abdelaziz Arada Izzedine, Abdelsalam Tidjani, Elisabeth Ngo Bum, Céline Lambert, Bruno Pereira, Jehan-François Desjeux, Vincent Sapin

**Affiliations:** 1 Institut National Supérieur des Sciences et Techniques d’Abéché (INSTA-Tchad), Abéché, Tchad; 2 Institut de Recherche en Élevage pour le Développement (IRED), N’Djamena, Tchad; 3 Biochimie et Biologie Moléculaire, CHU, Clermont-Ferrand, France; 4 Hôpital de la mère et de l’Enfant, Ndjaména, Tchad; 5 Faculté des Sciences de l’Université de Ngaoundéré, Ngaoundéré, Cameroun; 6 Faculté de Médecine, Université de Ndjaména, Ndjaména, Tchad; 7 Unité Biostatistiques, DRCI, CHU, Clermont-Ferrand, France; 8 Académie Nationale de Médecine, Paris, France; TNO, NETHERLANDS

## Abstract

**Background:**

Chad Lake is a central place in a region with a high prevalence of vitamin A deficiency. Spirulina, a natural source of β-carotene, is traditionally produced and eaten as “Dihé” around Chad Lake. β-carotene spirulina has been found to have a high conversion factor to retinol. The aim of the study was to assess if the retinol status between healthy women eating spirulina Dihé daily (SPI+) and not (SPI-) in the Chad Lake area was different.

**Methods:**

This study was observational: 88 healthy women were recruited and selected according to clinical criteria and their willingness to participate. They were divided in two groups according to their Dihé daily consumption: those who eat Dihé (SPI+; n = 35) and those who do not (SPI-; n = 35). After anthropometric and dietary assessments, blood retinol, β-carotene, retinol binding, and inflammatory/nutritional proteins were measured.

**Results:**

The diet between groups was identical, except for β-carotene consumption, which was higher in SPI+ than in SPI- (10.8 vs. 1.8 mg/day). The serum retinol and β-carotene concentrations were significantly higher in SPI+ than in SPI- at 1.26 ± 0.36 μmol/l versus 1.03 ± 0.31 μmol/l (p = 0.008) and 0.59 ±0.37 μmol/l versus 0.46± 0.31 μmol/l (p = 0.04), respectively. Seventy-seven percent of SPI+ versus 29% of SPI- had an adequate blood retinol value (p = 0.01).

**Conclusion:**

The results confirm that β-carotene in spirulina is an effective positive modulator of blood retinol status. Dihé is a potential natural source of β-carotene to achieve a proper vitamin A status in healthy women living near Chad Lake.

## Introduction

Vitamin A deficiency (VAD) is one of the most important causes of preventable childhood blindness and is a major contributor to morbidity and mortality from infections, especially in children and pregnant women; this condition affects the poorest segments of the population, particularly those in low and middle-income countries. The primary cause of VAD is the lack of adequate vitamin A intake. Its consequence is most apparent during stages of life with high nutritional needs (e.g., early childhood, pregnancy, and lactation). A variety of interventions are being used to improve the vitamin A status of populations, including dietary diversification, vitamin A or β-carotene supplementation, and fortification [1–6]. As of 2011, over 30 million people live in the Chad Basin, and the population continues to grow rapidly. The main economic activities in the area are farming, herding, and fishing. At least 40% of the rural population of Chad Basin live in poverty and routinely experience chronic food shortages. VAD, defined as plasma retinol < 0.70 μmol/l, is highly prevalent in the area [7]. This condition could be attributed to the low consumption of food rich in vitamin A, such as liver, vegetables, and fruits, particularly during the dry season [8; 9].

Spirulina is a photo-autotroph alga considered to be a valuable additional food source of some macro- and micronutrients, including carotenoids (mainly β-carotene) [10–12]. Spirulina, which is a natural source of β-carotene, is traditionally produced as “Dihé” around Chad Lake. It is commonly used by some locals as a powder condiment mixed in their main and only dish [13]. A human supplementation study showed that “Bitot’s spots,” a symptom of VAD, decreased from 80% to 10% after consumption of 1 g spirulina per day for ≥150 days in 5,000 preschool-age children in Chennai (India, another country with a high prevalence of VAD) [14]. In another study, a daily dose of β-carotene from 2 g spirulina was administered to 400 school children, and the results showed an increase in the children’s vitamin A status to the same level as those administered with pure vitamin A [15]. In a study in rural areas of China, the efficacy of spirulina in improving the total body vitamin A stores of school-age children when they consumed spirulina in their daily meals was assessed. After the 10-week intervention, the serum β-carotene concentrations of children who received a 2 or 4 g spirulina supplement increased by 0.160 and 0.389 μmol/l, respectively. Their total body vitamin A stores increased significantly, with a median increase of 0.160 and 0.279 mmol in children taking 2 and 4 g, respectively [15]. This beneficial effect is attributed to the high β-carotene conversion factor of spirulina [16]. In a group of 10 well-nourished Chinese men following a low vitamin A diet, 4.5 mg spirulina β-carotene consumed with 22 g fat was found to have the same vitamin A activity as that achieved with 1 mg retinol. This result suggested that 4.1 mg powder could meet the US recommended dietary allowance of 900 μg of retinol activity equivalents for men [17].

Thus, the aim of our study was to assess the retinol status of healthy women eating spirulina in the form of Dihé in the Chad Lake area. The examination of such a population is different from that conducted in previous experimental studies because a well-defined diet was supplemented with specially prepared spirulina. Our study does not experiment a supplementation or a fortification intervention; rather, it aims at exploring the long-term consequences of consuming spirulina as a local common food in healthy women. The selection of Chad Lake area as the study site enables the testing of the effect of Dihé consumption because not all families in the area eat Dihé, leading to the presence of both study groups in a similar geographical environment.

## Materials and methods

### Geographical site of the clinical investigation

Spirulina is naturally produced near the Chad Lake area, particularly in the Kanem and Lac regions. The dominant ethnic group in this region is the Kanembou, followed by the Arabs. Spirulina is collected and sun dried in a product called Dihé [13]. The final product is a thin green wafer. It is used as a powder mixed in the main dish; as such, it is used daily in some populations, especially among the Kanembou. It is poorly accepted in other populations, mostly the Arabs. As the Kanembou and Arabs have common food, except Dihé, the situation is conducive to studying the effects of Dihé on vitamin A status.

### Ethical issues and shipping of the serum samples

The research review and the ethical review committees of the National Ethical Committee of Chad (Comité National de Bioéthique du Tchad, CNBT) and Clermont Ferrand (Comité de Protection des Personnes Sud-Est) approved the study protocol. After a personal interview with the women in the local language, written informed consent was obtained from all 70 of them prior to their enrollment in the study. All women with overt disease (exclusion criterion) at screening were sent to Mao Hospital (Kanem region) for proper investigation and treatment. Once the plasma retinol concentrations of the women were known, everyone with VAD was treated according to standard international recommendations [7].

### Study design

**Fig 1:**
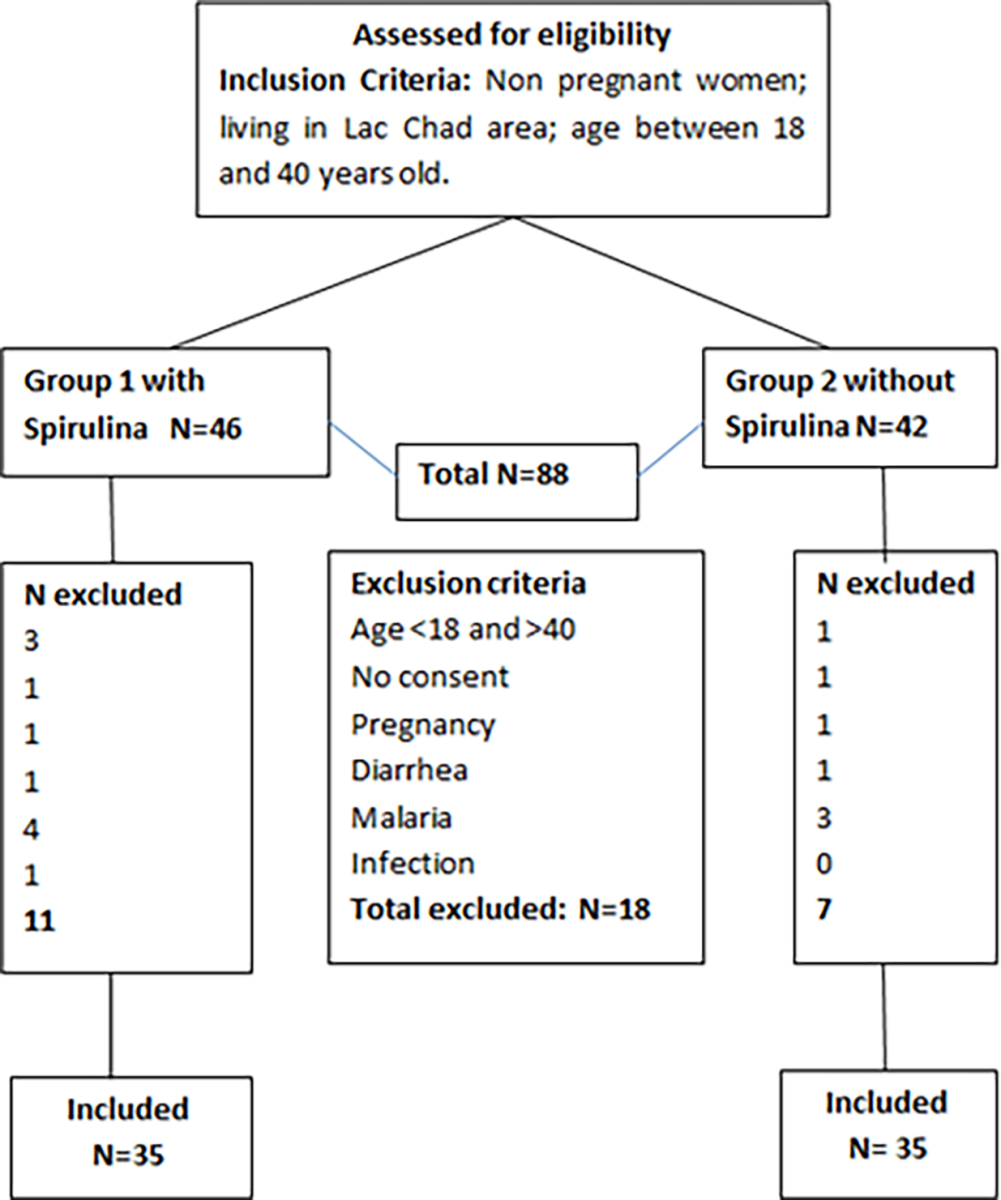
Flow chart of the enrolled women divided into the SPI+ and SPI- groups.

The field study was conducted in May 2016. It was restricted to the Kanem region because this area is well secured by the Chadian army against terrorist activities of Boko Haram. Eighty-eight women were first assessed for eligibility (Fig 1). The exclusion criteria were below 18 years or more than 40 years of age, did not consent to participate in the study, pregnancy, diarrhea, malaria, or infection (fever). The participants were divided in two groups according to their spirulina consumption, namely, those who eat Dihé (SPI+ (n = 35)) and those who do not (SPI- (n = 35)). Dihé consumption was defined as three times of Dihé consumption in the previous seven days. The other group did not consume Dihé in the previous week; in fact, they are reluctant to consume it. The individual participants’ daily food consumption was weighed (± 1g) prior to being mixed with spirulina (Table 1). As the diet was essentially the same in both groups except for spirulina, Table 1 presents a typical meal for women in both groups. The nutriment composition was analyzed using linear programming (NutriSurvey 2010, EBISpro, Germany) [18] with a minor modification in the food composition table to take into account the local food. The recommended values are for healthy 25-year-old women.

**Table 1:** Common daily food intake in the SPI- and SPI + groups. All data were similar in both groups, except the quantity of spirulina Dihé and the related amount of b-carotene.

**Food**		**Amount/day**	
Maize semolina		500 g	
Ogra		80 g	
Beef, minced and cooked		123 g	
Peanut oil		79 g	
Onions fresh		106 g	
Garlic fresh		21 g	
Drinking water		2000 g	
Quantity of spirulina Dihé consumed /person/day			
- in SPI+		9 g	
- in SPI-		0 g	
**Nutrient content**	**Analyzed value**	**Recommended value /day**	**Percentage fulfillment**
Energy	2770.6 kcal	2676.6 kcal	104%
Water	2309.1 g	2700.0 g	86%
Protein	81.5 g (12%)	79.1 g (12%)	103%
Fat	99.6 g (32%)	90.8 g (< 30%)	110%
Carbohydrates	383.3 g (56%)	382.1 g (> 55%)	100%
Dietary fiber	30.2 g	30.0 g	101%
Alcohol	0.0 g	-	-
PUFA	25.2 g	10.0 g	252%
Cholesterol	75.8 mg	-	-
Sodium	90.9 mg	2000.0 mg	5%
Potassium	1082.1 mg	3500.0 mg	31%
Calcium	177.8 mg	1000.0 mg	18%
Magnesium	169.1 mg	310.0 mg	55%
Phosphorus	662.1 mg	700.0 mg	95%
Iron	10.1 mg	15.0 mg	67%
Zinc	10.8 mg	7.0 mg	155%
Vitamin E (eq.)	14.2 mg	12.0 mg	118%
Vitamin B1	0.9 mg	1.0 mg	90%
Vitamin B2	0.6 mg	1.2 mg	49%
Vitamin B6	1.3 mg	1.2 mg	109%
Total Folic acid	64.1 μg	400.0 μg	16%
Vitamin C	122.8 mg	100.0 mg	123%
Vitamin A	389.7 μg	800.0 μg	49%
Approximate amount of β-carotene consumed person/day			
- in SPI+	10.8mg		
- in SPI-	1.8mg		

After anthropometric and dietary assessments, the women’s blood samples were collected. The serum was immediately frozen (−20°C) and shielded from the light and then sent to the Biochemistry laboratory of University Hospital of Clermont-Ferrand (France) for storage (−80°C) and subsequent analysis of retinol, β-carotene, and proteins (retinol binding protein [RBP], transthyretin (pre-albumin)/TTR, C-reactive protein [CRP], albumin, and orosomucoid).

### Biological measurements

Vitamin A and β-Carotene (in μmol/l) were extracted from the serum with Chromsystems kits and analyzed with HPLC (Prominence HPLC, SHIMADZU) following the standard method validated by the manufacturer (Chromsystems Instruments & Chemicals GmbH, Germany). Serum proteins concentrations (RBP, TTR, CRP, albumin, and orosomucoid) were assessed with the immuno-nephelemetric method on a Dimension VISTA analyzer by using the methods and reagents recommended by the in-vitro diagnosis society Siemens Health Care (Marburg/Germany). The prognosis inflammatory and nutritional index (PINI) was calculated utilizing the original formula that considered blood concentrations of TTR, CRP, albumin, and orosomucoid [19].

### Statistical analysis

Because of the novelty of our research, estimating an optimal sample size appeared difficult for the main objective of this study, which was to assess the retinol status in healthy women eating spirulina in the form of Dihé. Therefore, the sample size was estimated according to Cohen’s recommendations, which defined effect-size bounds as small (ES: 0.2), medium (ES: 0.5), and large (ES: 0.8, “grossly perceptible and therefore large”). We calculated that 33 women per group would be adequate in highlighting an effect size equal to 0.8 for a two-sided type-I error set at α = 0.05 and a statistical power of 90%. Considering possible missing data, we finally chose to include a minimum of 35 women per group (see Fig 1).

Statistical analysis was performed using Stata software (version 13, StataCorp, College Station, Texas, US). The tests were two sided, with a type-I error set at α = 0.05. Baseline characteristics were presented as mean ± standard-deviation according to the statistical distribution for continuous data (normality studied using the Shapiro-Wilk test) and as the number of women and associated percentages for categorical parameters. The Kolmogorov-Smirnov test was applied to compare the statistical distribution of vitamin A and β-carotene between the SPI+ and SPI- groups. Then, comparisons between independent groups (SPI+ and SPI-) were performed with the Student’s t-test or the Mann-Whitney test, if t-test conditions could not be respected (normality and homoscedasticity analyzed with the Fisher-Snedecor distribution). The results were expressed as effect size, 95% confidence interval, and p-value. The comparisons for categorical parameters were performed with a chi-squared test or, if necessary, Fisher’s exact test. The analysis of the relationships between quantitative parameters was performed using correlation coefficients (Pearson or Spearman, according to statistical distribution) and represented graphically with a color-coded heatmap. Finally, multivariate analyses were performed to consider adjustment on possible confounding factors, such as body mass index (BMI). Linear multiple regressions were applied for continuous outcomes and logistic models for dichotomous endpoints.

## Results

As illustrated in Table 1, the food pattern in both groups was very similar. As expected, the diet was monotonous on a daily basis. The amount of food was estimated at the meal time in which there is a common plate for the family. In agreement with the anthropometric characteristics, the macronutrient content of the food was within the recommended values per day. The main difference between groups was the beta-carotene content: 1.8 and 10.8 mg (Table 1) in the SPI- and SPI+ groups, respectively.

The anthropometric characteristics of the two groups of non-pregnant women (SPI- and SPI+) are described in Table 2. Statistical differences were observed between groups for weight and BMI, which were both significantly lower in the SPI- group. This result agrees with the common finding that VAD is often associated with malnutrition [20; 21]. Nevertheless, the mean BMI values in both groups were within reference values [22], excluding severe malnutrition, as confirmed by the higher albumin concentrations than the alert blood values, not significantly different between both groups (Table 3). Note that the other proteins (TTR, CRP, albumin, and orosomucoid) were also statistically similar to the PINI score.

**Table 2:** Anthropometric characteristics of non-pregnant women consuming spirulina (SPI+) and non-pregnant women not consuming spirulina (SPI-).

	SPI + (n = 35)	SPI - (n = 35)	p value
**Age (years)**	25.8 ± 4.8	26.1 ± 5.9	0.81
**Weight (kg)**	62.9 ± 4.2	57.8 ± 4.65	<0.001
**Height (m)**	1.67 ± 0.05	1.67±0.04	0.88
**Body Mass Index (kg/m2)**	22.69 ± 1.63	20.81±1.84	<0.001

**Table 3:** Biological parameters of non-pregnant women consuming spirulina (SPI+) and non-pregnant women not consuming spirulina (SPI-).

	SPI +	SPI -	P univariate	Effect-size [95%CI]	p multivariate^1^
Vitamin A (μmol/l), mean ± sd	1.26 ± 0.36	1.03 ± 0.31	0.006	0.68 [0.20; 1.16]	0.008
Vitamin A < 1.05 μmol/l, n (%)	10 (28.6)	20 (57.1)	0.02	/	0.01
β-carotene (μmol/l), mean ± sd	0.59 ± 0.37	0.46 ± 0.31	0.11	0.39 [−0.09; 0.86]	0.04
RBP (mg/l), mean ± sd	33.24 ± 10.57	27.40 ± 7.11	0.01	0.65 [0.16; 1.13]	0.006
TTR (mg/l), mean ± sd	0.23 ± 0.05	0.21 ± 0.08	0.44	0.19 [−0.28; 0.66]	0.47
Albumin (g/l), mean ± sd	42.33 ± 3.56	41.84 ± 4.23	0.60	0.13 [−0.34; 0.59]	0.44
CRP (mg/l), mean ± sd	3.32 ± 1.63	4.01 ± 2.46	0.17	0.33 [−0.80; 0.14]	0.17
Orosomucoid (mg/l), mean ± sd	0.87 ± 0.24	0.86 ± 0.26	0.81	0.06 [−0.41; 0.53]	0.82
Ratio retinol/RBP, mean ± sd	0.83 ± 0.25	0.82 ± 0.27	0.82	0.05 [−0.42; 0.52]	0.10
Ratio RBP/TTR, mean ± sd	0.39 ± 0.10	0.35 ± 0.09	0.15	0.35 [−0.13; 0.82]	0.13
Ratio retinol/ TTR, mean ± sd	0.31 ± 0.09	0.28 ± 0.09	0.11	0.38 [−0.09; 0.86]	0.47
PINI, median [IQR]	0.26 [0.20; 0.39]	0.29 [0.22; 0.41]	0.32	−0.34 [−0.81; 0.13]	0.17

^1)^ Multivariate analyses were performed to consider adjustment on possible confounding factors as BMI

**Fig 2:**
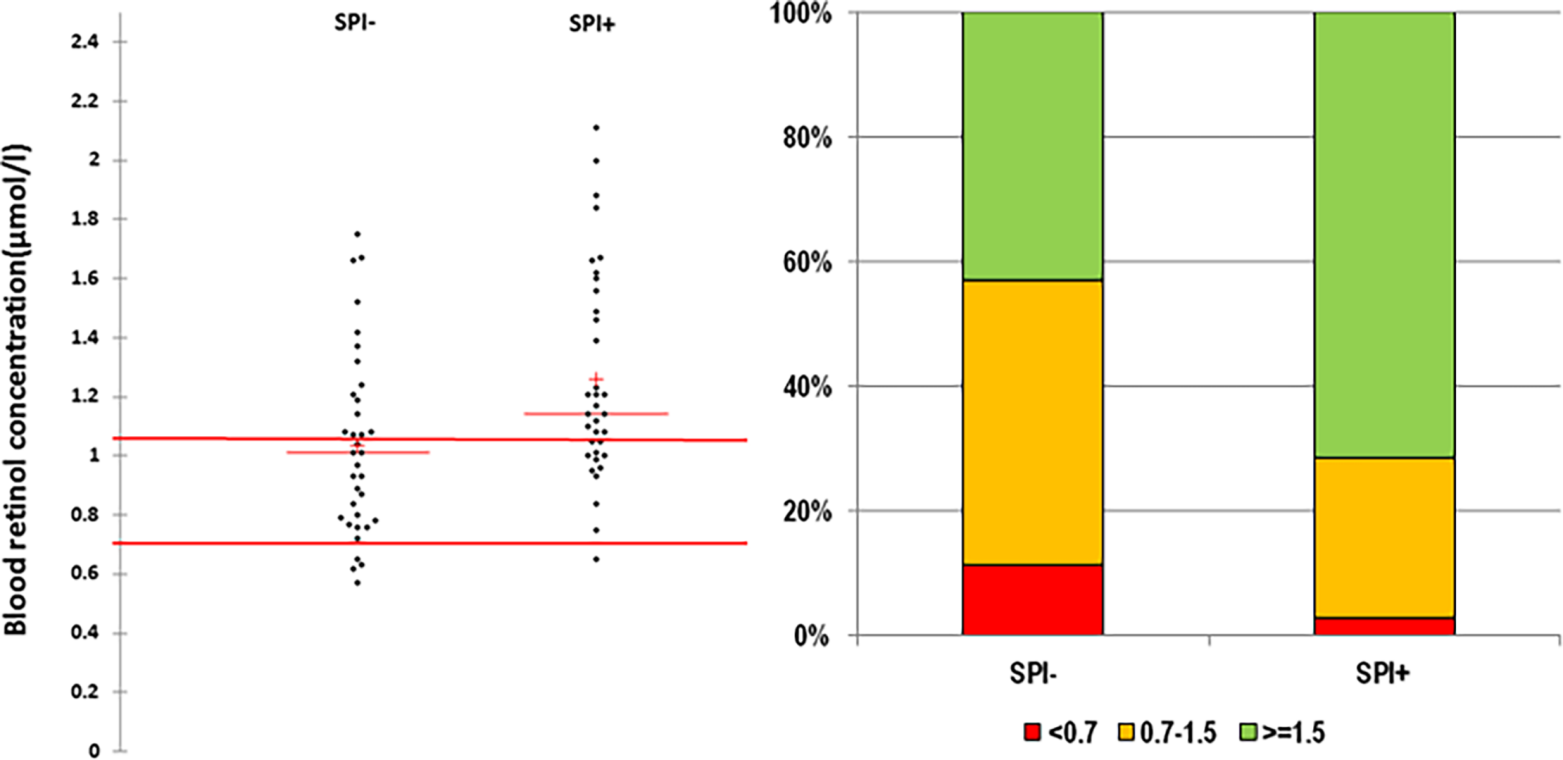
Retinol blood concentrations and distribution in the SPI- and SPI+ groups. The left panel presents all individuals’ blood values, with two red horizontal bars representing the higher value, the adequate blood level of retinol (1.05 μmol/l), and the lower one, which is the cut-off to define a VAD biologically (0.7 μmol/l). The right panel represents the repartition of both groups by using the adequate and VAD blood levels.

**Fig 3:**
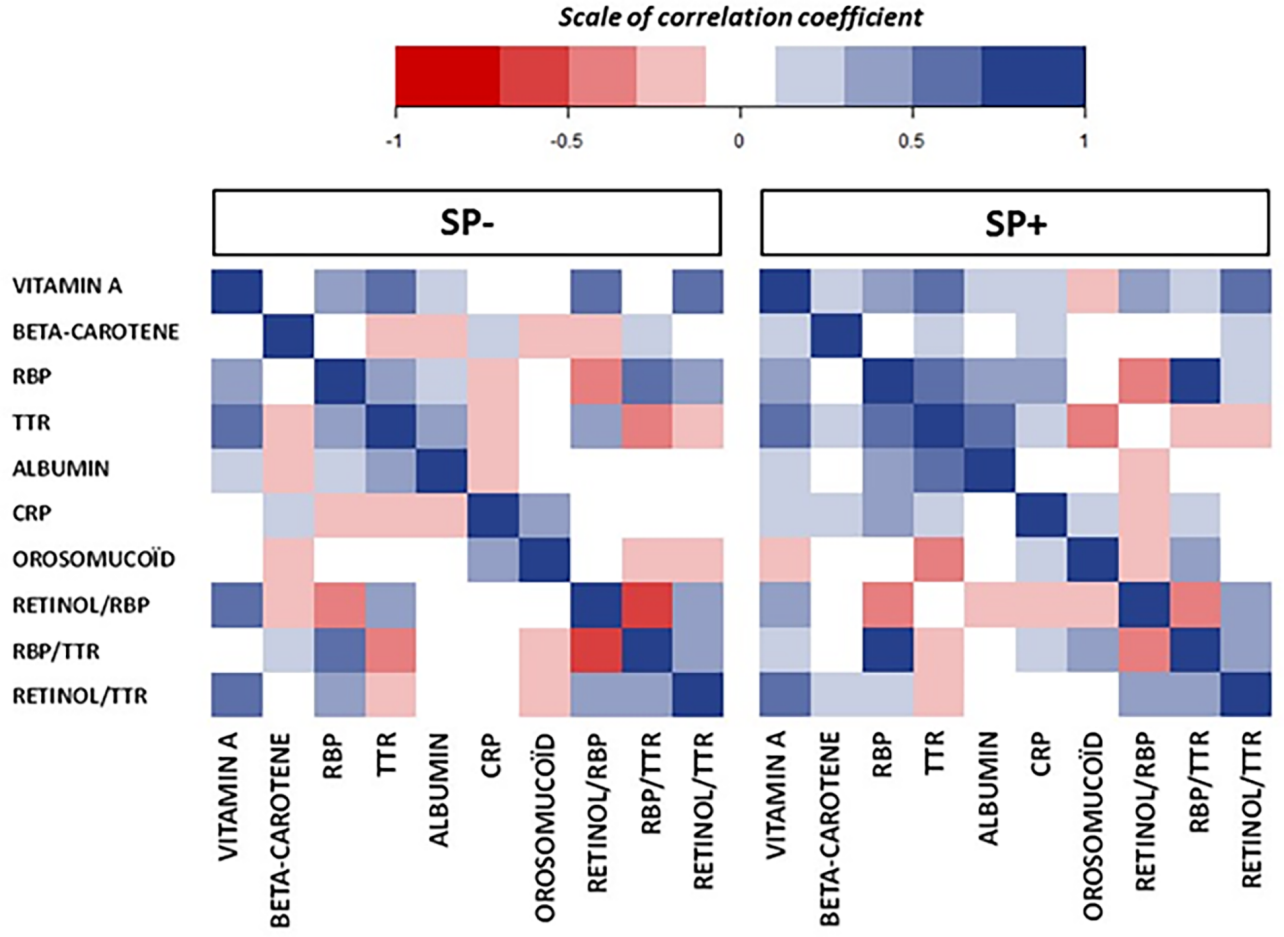
Correlations between biological parameters in the SPI- and SPI+ groups.

The serum retinol (vitamin A) concentrations of both groups of women are presented in Table 3 and Fig 2. The retinol serum concentration is significantly different (p = 0.008) between the two groups, with an average of 1.26 (± 0.36) μmol/l in the SPI+ group and 1.03 (± 0.31) μmol/l in the SPI- group. As the specific retinol carrier protein, RBP also had lower concentrations in the SPI- group than in the SPI+ group (p = 0.006), leading to the similar retinol/RBP ratio of the two groups. The presence of a ternary complex (RBP-TTR-Rol) was also illustrated by the similar correlation coefficient between vitamin A-RBP, vitamin A-RBP ratio, and vitamin A-TTR of both groups (Fig 3). Using the well-established cut-off of 1.05 μmol/l as recommended blood retinol level, we clearly established that the percentage of retinol’ adequate status was higher in SPI+ than SPI- group (p = 0.01). In addition, the percentage of women with biological VAD deficiency (<0.7 μmol/l) was higher in the SPI- group (11.4%) than in the SPI+ group (2.9%). Altogether, the results described SPI+ women as a group presenting a more adequate biological retinol status than the SPI- women.

**Fig 4:**
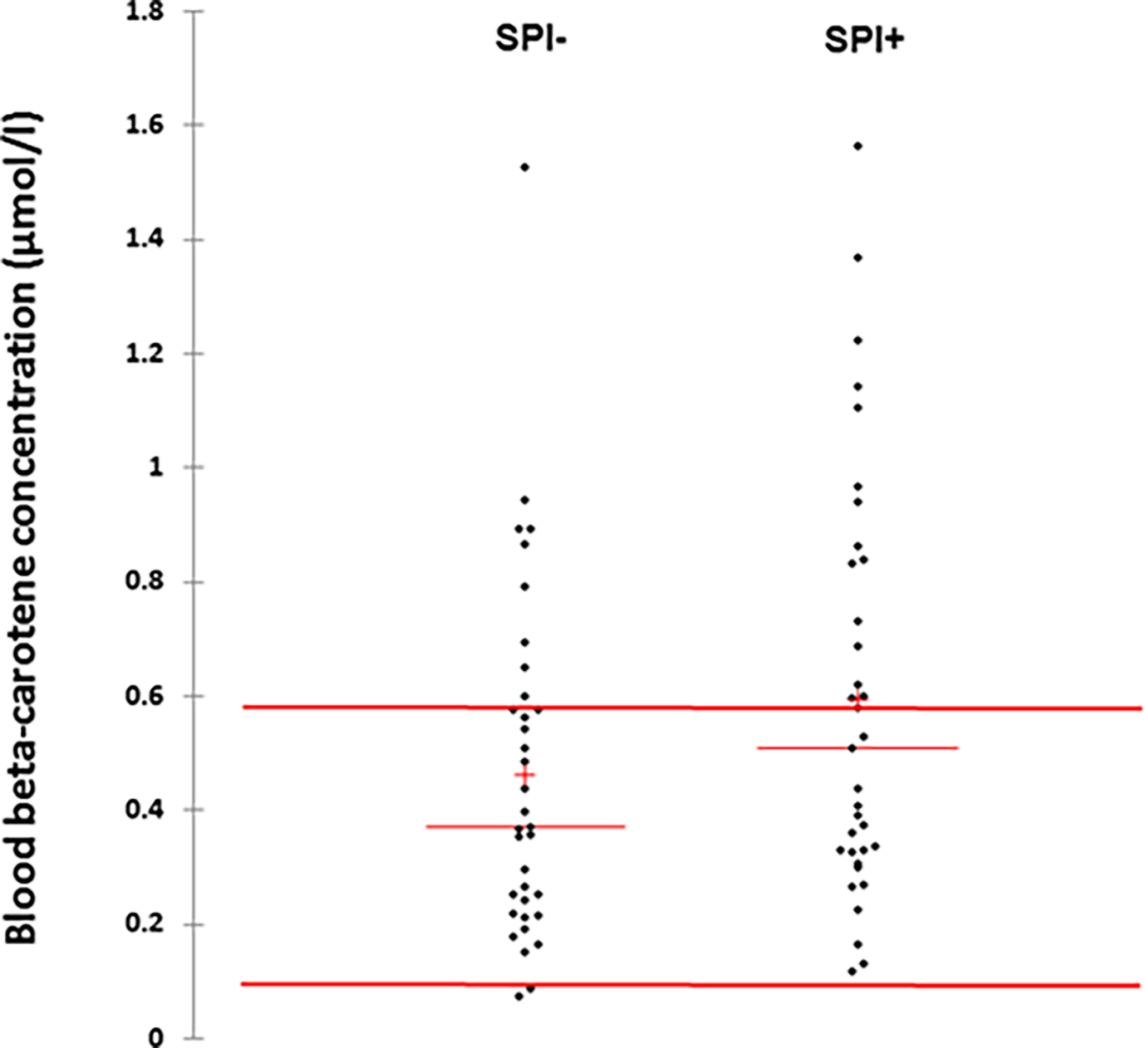
β-carotene blood concentrations and distribution in the SPI- and SPI+ groups. All individuals’ blood values were presented for both groups, with two horizontal bars representing the reference ranges used in the laboratory (0.074–0.599 μmol/l).

As indicated in Table 3, the β-carotene blood levels are more elevated in the SPI+ group than in the SPI- group (p = 0.04), with an average of 0.59 (± 0.37) and 0.46 (± 0.31) μmol/l respectively; included in the reference ranges of the laboratory (0.074–0.599 μmol/l). As illustrated in Fig 4, the percentage of upper concentration than 0.599 μmol/l was higher in the SPI+ group than in the SPI- group (43.3% vs. 26.7%). However, we could not find retinol values that are more than 2.45 μmol/l in both SPI+ and SPI- groups, which could be considered the biological stigmata of hypervitaminosis A. A stronger correlation coefficient was found between vitamin A and β-carotene (Fig 3) in SPI+, suggesting the link between both parameters.

## Discussion

Our results confirm that β-carotene in spirulina is an effective precursor of retinol [23; 15; 24]. They further suggest that Dihé is a potential natural dietary source of β-carotene to achieve an adequate retinol status in healthy women living near Chad Lake. In previous studies, similar results were obtained but in acute conditions designed as clinical trials that were conducted within a short period of time. The originality of our work is that we did not change the participants’ usual consumption behavior and diet content, as spirulina was regularly consumed as a common condiment in the meals of the SPI+ group. Our work was based on the observation of continuous real life of β-carotene consumption. In this case, differences in blood β-carotene concentrations between both groups is attempted, regards to the daily ingested quantity (10.8 mg) by SPI+ one. Indeed, this concentration was previously demonstrated to be altered by eating a meal containing ≥ 6 mg of β-carotene [25]. In contrast to that of previous studies, the design of our work establishes the conversion of β-carotene into vitamin A by assessing the positive changes in serum retinol after regular consumption with β-carotene of spirulina because it is the single difference between both groups studied. In our opinion, this concept is clearly established in our results, even if we used a more rustic approach than those based on the stable isotope reference method with labeled synthetic β-carotene.

The prevention of VAD is still a matter of debate [2–6]. Several interventions to prevent VAD used bioengineered food, such as golden rice or high-β-carotene yellow maize [1; 8; 26–31]. Their results based on retinol plasma level are encouraging but are not entirely convincing. In addition, bio-fortification has technical limits, including cost and regular supply. Another public policy to control VAD is to avoid such interventions if a normal vitamin A status can be sustained from a diversified diet. In our study, Dihé is not bio-fortified with β-carotene and is available all year round as local food. An additional advantage of β-carotene supplementation with spirulina is the homeostatic and strict control of its intestinal absorption and enzymatic conversion into retinol. This advantage was verified in the SPI+ group through the absence of retinol blood values that increase to hyper-vitaminosis A alert concentrations, thereby strengthening the possibility of using this supplementation in pregnant women. Pregnancy is a physiological period with a high risk of VAD, which leads to fetal developmental abnormalities. Natural consumption without hyper-vitaminosis A risk needs to be checked in future studies.

In the group consuming spirulina, the blood retinol and β-carotene concentrations presented a significant variation between women and did not reach reference values in all women. For β-carotene, it may be due to several factors: the women may have consumed less than 9 g spirulina every day, as their dietary intake was not precisely assessed by direct weighing. Alternatively, it may also be related to intestinal absorption [10; 32]. Common meals contain 79 g peanut oil, with mostly oleic, linoleic, and palmitic acid, a condition representing the optimal absorption of β-carotene [33]. Spirulina has been shown to contain major proportions of mono-and galactosyl diglyceride, sulfo-quinovosyl diglyceride, and phosphatidyl glycerol, but none possess lecithin, phophatidyl ethanolamine, or phosphatidyl inositol. However, the functional role of β-carotene absorption is not yet known [34]. Specific individual conversion rates of β-carotene into retinol could be proposed to explain retinol variations.

Some limitations of our study could be noted. First, the vitamin A status of the women was determined from plasma retinol concentrations and not through the measurement of total body store or from a dose-response test [35]. Such explorations would be very difficult to conduct in the Chad context. In addition, the used indicator of VAD (blood level < 0.70 μmol/l) was prescribed by the WHO. Nevertheless, our concordant biological data are in agreement with the VAD status as for example, the reduction more than the destabilization of the circulatory complex of vitamin A in the SPI- group. The second limitation of our study was that we don’t try to identify the clinical consequences of VAD. It was originally set, but the unsafe area under Boko Haram threat made the realization of this target difficult. Nevertheless, this limitation does not alter the primary conclusion of our study regarding the effect of Dihé on vitamin A status. The third limitation is the food habits identified in the two ethnic groups (Kanembou versus Arabic), which could have genetic differences in their global metabolism of carotenoids and retinoids. Our main inclusion criterion was that the women had to be healthy, thereby removing clinical situations known to alter β-carotene bioavailability [36]. Infectious diseases, including infectious diarrhea, have been well demonstrated to function as limiting factors to β-carotene absorption [37]. Our results should be tested in such populations in future studies.

## Conclusion

We demonstrate that β-carotene in spirulina is an effective positive modulator of blood retinol status and that a natural food compound, Dihè, is a potential natural source of β-carotene to achieve an optimal vitamin A status in healthy women living near Chad Lake. These initial results provide evidence that may encourage future studies to test this strategy on pregnant or unhealthy women.

## References

[1] Tang G, Qin J, Dolnikowski GG, Russell RM, Grusak MA. Golden Rice is an effective source of vitamin A. Am.J.Clin.Nutr. 2009; 89:1776–1783. doi: 10.3945/ajcn.2008.27119 19369372 10.3945/ajcn.2008.27119 PMC2682994

[2] Benn CS, Aaby P, Arts RJ, Jensen KJ, Netea MG, Fisker AB. An enigma: why vitamin A supplementation does not always reduce mortality even though vitamin A deficiency is associated with increased mortality. Int J.Epidemiol. 2015; 44: 906–918. doi: 10.1093/ije/dyv117 26142161 10.1093/ije/dyv117 PMC4521135

[3] Mason J, Greiner T, Shrimpton R, Sanders D, Yukich J. Response to: Letter to the editor by Benn C, Fisker A and Aaby P. Int J.Epidemiol. 2015a; 44: 367–368. doi: 10.1093/ije/dyu266 25586994 10.1093/ije/dyu266

[4] Mason J, Greiner T, Shrimpton R, Sanders D, Yukich J. Vitamin A policies need rethinking. Int J.Epidemiol. 2015b; 44: 283–292. doi: 10.1093/ije/dyu194 25306559 10.1093/ije/dyu194

[5] Mason JB, Sanders D, Greiner T, Shrimpton R, Yukich J. Vitamin A deficiency: policy implications of estimates of trends and mortality in children. Lancet Glob.Health. 2016; 4, e21 doi: 10.1016/S2214-109X(15)00246-6 26718802 10.1016/S2214-109X(15)00246-6

[6] West KP Jr, Sommer A, Palmer A, Schultink W, Habicht JP. Commentary: Vitamin A policies need rethinking. Int J.Epidemiol. 2015; 44: 292–294. doi: 10.1093/ije/dyu275 25617646 10.1093/ije/dyu275

[7] Vitamin and Mineral Nutrition Information System (VMNIS); Micronutrients database; WHO Global Database on Vitamin A Deficiency, Vitamin A deficiency data by country. WHO 2017 Available from: http://www.who.int/vmnis/database/vitamina/countries/en/

[8] Augusto RA, Cobayashi F, Cardoso MA. Associations between low consumption of fruits and vegetables and nutritional deficiencies in Brazilian schoolchildren. Public Health Nutr. 2014, 1–10. 24963861 10.1017/S1368980014001244 PMC10271615

[9] West KP Jr, Mehra S. Vitamin A intake and status in populations facing economic stress. J.Nutr. 2010; 140: 201S–207S. doi: 10.3945/jn.109.112730 19939993 10.3945/jn.109.112730

[10] Ciferri O. SPIrulina, the edible microorganism. Microbiol.Rev. 1983; 47: 551–578. 6420655 10.1128/mr.47.4.551-578.1983 PMC283708

[11] El-Baky HH, El Baz FK, El-Baroty GS. SPIrulina speciesnas a source of carotenoids and á-tocopherol and its anticarcinoman factors. Biotechnology. 2003; 2: 222–240.

[12] Gireesh T, Nair PP, Sudhakaran PR. Studies on the bioavailability of the provitamin A carotenoid, beta-carotene, using human exfoliated colonic epithelial cells. Br.J.Nutr. 2004; 92: 241–245. doi: 10.1079/BJN20041175 15333155 10.1079/BJN20041175

[13] GCP/CHD/029/EC: Projet pilote de développement de la filière « Dihé » au Tchad. Le développement de la filière spiruline ou « dihé » au service de la population et des personnes vulnérables au Tchad. FAO, 2016. Available from: http://www.fao.org/uploads/media/dihe_techdoc.pdf

[14] Seshadri CV. Large Scale Nutritional Supplementation with SPIrulina Alga. All India Coordinated Project on SPIrulina. 1993 Madras, India: Shri Amm Murugappa Chettiar Research Center.

[15] Li L, Zhao X, Wang J, Muzhingi T, Suter PM, Tang G, et al Spirulina can increase total-body vitamin A stores of Chinese school-age children as determined by a paired isotope dilution technique. J.Nutr.Sci. 2012; 1, e19 doi: 10.1017/jns.2012.21 25191548 10.1017/jns.2012.21 PMC4153073

[16] Tang G. Bioconversion of dietary provitamin A carotenoids to vitamin A in humans. Am.J.Clin.Nutr. 2010; 91: 1468S–1473S. doi: 10.3945/ajcn.2010.28674G 20200262 10.3945/ajcn.2010.28674G PMC2854912

[17] Wang Z, Yin S, Zhao X, Russell RM, Tang G. beta-Carotene-vitamin A equivalence in Chinese adults assessed by an isotope dilution technique. Br.J.Nutr. 2004; 91: 121–131. 14748945 10.1079/bjn20031030

[18] Darmon N, Fergusson E, Briend A. Linear and non linear programming to optimize the nutrient density of a population'sdiet: an example based on diets of preschool children in rural Malawi. Am. J. Clin. Nutr.2002; 75:245–253. 11815314 10.1093/ajcn/75.2.245

[19] Ingenbleek Y, Carpentier YA. A pronostic inflammatory and nutritional index scoring critically ill patients. Int J Vit Nutr Res 1985; 55: 91–101. 3922909

[20] WHO. Global prevalence of vitamin A deficiency in populations at risk 1995–2005 WHO Global Database on Vitamin A Deficiency. 2009; World Health Organisation, Geneva.

[21] Wiseman EM, Dadon SB, Reifen R. The vicious cycle of vitamin A deficiency: A review, Critical Reviews in Food Science and Nutrition. 2016; doi: 10.1080/10408398.2016.1160362 27128154 10.1080/10408398.2016.1160362

[22] World Health Organization WHO, “BMI Classification”, 2012. Available from: http://apps.who.int/bmi/index.jsp?introPage=intro_3.html.

[23] Annapurna VV, Deosthale YG, Bamji MS. Spirulina as a source of vitamin A. Plant Foods Hum.Nutr. 1991; 41: 125–134. 1906616 10.1007/BF02194081

[24] Wang J, Wang Y, Wang Z, Li L, Qin J, Lai W, et al Vitamin A equivalence of Spirulina beta-carotene in Chinese adults as assessed by using a stable-isotope reference method. Am.J.Clin.Nutr. 2008; 87: 1730–1737. 18541562 10.1093/ajcn/87.6.1730

[25] Falquet J and Hurri JP. Spiruline: aspects nutrtionnels. Antenna Technologie 2006 (41 pages). https://www.antenna-france.org//wp-content/uploads/2014/06/spiruline-aspects-nutritionnels.pdf

[26] Tang G, Hu Y, Yin SA, Wang Y, Dallal GE, Grusak MA, et al beta-Carotene in Golden Rice is as good as beta-carotene in oil at providing vitamin A to children. Am.J.Clin.Nutr. 2012; 96: 658–664. doi: 10.3945/ajcn.111.030775 22854406 10.3945/ajcn.111.030775 PMC3417220

[27] La Frano MR, Woodhouse LR, Burnett DJ, Burri BJ. Biofortified cassava increases beta-carotene and vitamin A concentrations in the TAG-rich plasma layer of American women. Br.J.Nutr. 2013; 110: 310–320. doi: 10.1017/S0007114512005004 23332040 10.1017/S0007114512005004

[28] Muzhingi T, Gadaga TH, Siwela AH, Grusak MA, Russell RM, Tang G. Yellow maize with high beta-carotene is an effective source of vitamin A in healthy Zimbabwean men. Am.J.Clin.Nutr. 2011; 94: 510–519. doi: 10.3945/ajcn.110.006486 21715509 10.3945/ajcn.110.006486 PMC3142725

[29] Palmer AC, Healy K, Barffour MA, Siamusantu W, Chileshe J, Schulze KJ, et al Provitamin A Carotenoid-Biofortified Maize Consumption Increases Pupillary Responsiveness among Zambian Children in a Randomized Controlled Trial. J.Nutr. 2016a. 10.3945/jn.116.239202 27798345

[30] Palmer AC, Siamusantu W, Chileshe J, Schulze KJ, Barffour M, Craft NE, et al Provitamin A-biofortified maize increases serum beta-carotene, but not retinol, in marginally nourished children: a cluster-randomized trial in rural Zambia. Am.J.Clin.Nutr. 2016b;104: 181–190. doi: 10.3945/ajcn.116.132571 27169838 10.3945/ajcn.116.132571

[31] Talsma EF, Brouwer ID, Verhoef H, Mbera GN, Mwangi AM, Demir AY, et al Biofortified yellow cassava and vitamin A status of Kenyan children: a randomized controlled trial. Am.J.Clin.Nutr. 2016; 103: 258–267. doi: 10.3945/ajcn.114.100164 26675768 10.3945/ajcn.114.100164

[32] Yeum KJ, Russell RM. Carotenoid bioavailability and bioconversion. Annu Rev Nutr. 2002; 22:483–504. doi: 10.1146/annurev.nutr.22.010402.102834 12055355 10.1146/annurev.nutr.22.010402.102834

[33] Nagao A, Kotake-Nara E, Hase M. Effects of fats and oils on the bioaccessibility of carotenoids and vitamin E in vegetables. Biosci. Biotechnol. Biochem. 2013; 77: 1055–1060. doi: 10.1271/bbb.130025 23649270 10.1271/bbb.130025

[34] Nichols BW, Wood BJB. The occurrence and biosynthesis of gamma-linolenic acid in a blue-green alga, Spirulina platensis. Lipids. 1968; 3: 46 doi: 10.1007/BF02530968 17805841 10.1007/BF02530968

[35] Tanumihardjo SA, Permaesih D, Muhilal. Vitamin A status and hemoglobin concentrations are improved in Indonesian children with vitamin A and deworming interventions. Eur J Clin Nutr. 2004 9;58(9):1223–30. doi: 10.1038/sj.ejcn.1601953 15054437 10.1038/sj.ejcn.1601953

[36] Grune T, Lietz G, Palou A, Ross AC, Stahl W, Tang G, et al Beta-carotene is an important vitamin A source for humans. J.Nutr. 2010; 140: 2268S–2285S. doi: 10.3945/jn.109.119024 20980645 10.3945/jn.109.119024 PMC3139236

[37] Sommer A. Vitamin A supplementation and childhood morbidity. Lancet. 1993; 342: 1420. 7901697

